# Health care utilization in young adults with childhood physical disabilities: a nationally representative prospective cohort study

**DOI:** 10.1186/s12887-022-03563-0

**Published:** 2022-08-25

**Authors:** Kirkpatrick B. Fergus, Alan Zambeli-Ljepović, Lindsay A. Hampson, Hillary L. Copp, Jason M. Nagata

**Affiliations:** 1 Department of Surgery, University of California-San Francisco, San Francisco, CA USA; 2 Department of Urology, University of California-San Francisco, San Francisco, CA USA; 3 Department of Pediatrics, University of California-San Francisco, 550 16th Street, 4th Floor, Box 0530, San Francisco, CA 94143 USA

**Keywords:** Disability, Health care utilization, Health care transition, Emergency department, Hospitalization, Health disparity

## Abstract

**Background:**

Young people with physical disabilities face barriers to accessing health care; however, few studies have followed adolescents with physical disabilities longitudinally through the transition of care into adulthood. The objective of this study was to investigate differences in health care utilization between adolescents with physical disabilities and those without during the transition period from adolescent to adult care.

**Methods:**

We utilized the National Longitudinal Study of Adolescent to Adult Health, a prospective cohort study following adolescents ages 11–18 at baseline (1994–1995) through adulthood. Baseline physical disability status was defined as difficulty using limbs, using assistive devices or braces, or having an artificial limb; controls met none of these criteria. Health care utilization outcomes were measured seven years after baseline (ages 18–26). These included yearly physical check-ups, unmet health care needs, and utilization of last-resort medical care, such as emergency departments, inpatient hospital wards, and inpatient mental health facilities. Multiple logistic regression models were used to predict health care utilization, controlling for age, sex, race/ethnicity, insurance status, and history of depression.

**Results:**

Thirteen thousand four hundred thirty-six participants met inclusion criteria, including 4.2% with a physical disability and 95.8% without. Half (50%) of the sample were women, and the average age at baseline was 15.9 years (SE = 0.12). In logistic regression models, those with a disability had higher odds of unmet health care needs in the past year (Odds Ratio (OR) 1.41 95% CI 1.07–1.87), two or more emergency department visits in the past five years (OR 1.34 95% CI 1.06–1.70), and any hospitalizations in the past five years (OR 1.36 95% CI 1.07–1.72). No statistically significant differences in preventive yearly check-ups or admission to mental health facilities were noted.

**Conclusions:**

Young adults with physical disabilities are at higher risk of having unmet health care needs and using last-resort health care services compared to their non-disabled peers.

**Supplementary Information:**

The online version contains supplementary material available at 10.1186/s12887-022-03563-0.

Nationally, up to three percent of children [1] and about one in seven adults [2] live with a physical disability. Adolescents in this group face the dual challenge of transitioning from pediatric to adult health care teams and finding providers who will adequately address their disability without neglecting routine health maintenance. The pediatric to adult health care transition is often strained because adult providers report insufficient training for childhood conditions, and patients feel unprepared or abruptly transitioned [3]. Unstructured transitions are associated with poorer mental well-being, lower medication adherence rates, and higher costs of health care [4].

Physical disability exposes patients to a separate set of systemic inequities: poor coordination among physicians, inadequate resources for transportation and physical access to health care facilities, and feelings of neglect and isolation [4–8]. Each of these is amplified during the critical transition from pediatric to adult care [9], as young people with disabilities end up with insufficient preventive screening [10, 11], unmet health care needs [8, 12], and worse chronic disease health outcomes [10, 13] relative to the general population.

This health care gap has spurred numerous national and international transition guidance statements [4, 14–16] and targeted interventions for persons with disabilities or chronic health conditions [17–20]. Yet the most successful interventions yielded only modest and transient improvements in quality of life [21]. Furthermore, published studies examine diabetes, rheumatologic disease, and cystic fibrosis [22, 23] – diseases that rarely have primary physical implications. Of the studies centered on physical disabilities, few have followed adolescents longitudinally through the transition of care and into adulthood, though some have followed adults prospectively [8, 10, 12, 13].

This study uses a large, nationally representative prospective cohort study of adolescents to compare health care utilization outcomes between those with a physical disability and those without, over a seven-year period. We hypothesize that, compared to young adults without physical disabilities, those with physical disabilities will more frequently use health care resources of last resort, including emergency department (ED) visits and admissions to a hospital or mental health facility.

## Methods

### Study population

We used the National Longitudinal Study of Adolescent to Adult Health, a prospective cohort study following adolescents through adulthood over five data collection waves. For this specific analysis, we analyzed data from baseline ages 11–18 (Wave I, 1994–1995, when exposures were measured) through young adulthood to ages 18–26 (Wave III, 2001–2002, when outcomes for this study were measured). This nationally representative sample was collected from 80 US high schools with paired middle schools, selected based on region, urbanicity, size, type and ethnicity. Further study design details are described elsewhere [24]. Of the 15,197 participants measured at Wave III follow-up (ages 18–26), 44 were excluded due to no baseline disability status. We further excluded participants that did not have health care utilization data (*n* = 619), and those with missing covariates (*n* = 1,118), resulting in a total cohort of 13,416 participants. This cohort study and its procedures were approved by the University of North Carolina Institutional Review Board.

### Physical disability status

Baseline physical disability status was defined by one or more of the following criteria: 1) difficulty using limbs such as “hands, arms, legs or feet;” 2) using assistive devices such as a “cane, walker, medically prescribed shoes, wheelchair or scooter” to get around; and 3) using a “brace” or “artificial limb” for a “hand, arm, leg or foot.” This definition of physical disability is similar to prior studies such as that of Cheng and Udry in 2002 [25]. Refer to Additional file 1: Appendix Table A for the questions used to determine physical disability status and to define covariates. Only adolescents (ages 11–18) reporting physical disability status at baseline in 1994–95 were considered physically disabled in our study. The comparison group of people without physical disabilities reported no to all three questions.

### Health care utilization

Health care utilization outcomes were measured seven years after baseline (Wave III, ages 18–26). We investigated if participants were engaged in preventive medical care, defining this variable as the attendance of a routine physician check-up in the last year (yes/no). We measured unmet health care needs using the question, “*Has there been any time in the past 12 months when you thought you should get medical care, but you did not?*” Significant ED usage was defined as the above average visitation to the ED (equivalent to at least two ED visits based on the average at the time of data collection) in the past five years (yes/no). This method had been used previously [26], and we considered the threshold of “above average” to be clinically significant. Furthermore, our results were robust to a sensitivity analysis performed using the log-transformed number of ED visits. Other health care utilization variables included hospitalization in the past five years (yes/no) and mental health facility admission in the past five years (yes/no).

### Covariates

Age, sex, and race/ethnicity were based on baseline (Wave I) self-report. Household income was based on parents’ response at baseline to the question: “*About how much total income, before taxes did your family receive in 1994? Include your own income, the income of everyone else in your household, and income from welfare benefits, dividends, and all other sources*.” This variable was intended to be a proxy for socio-economic status. Gaussian normal regression imputation models were used to impute income for the 1,638 parents who either refused to answer the income question or stated they did not know based on race/ethnicity, region, hours of self-reported work per week by mother and father, and parental marital status, created by the authors similar to the method used in previous studies [27, 28]. Highest parent education was based on parents' baseline response regarding the highest educational attainment for themselves or their spouse/partner (whichever was higher). Responses were dichotomized into high school or less versus more than high school, similar to other large population-based studies [29]. Additional chronic health conditions used in the model include depression, asthma, diabetes, and history of seizure disorder, all self-reported and measured in Wave III [30, 31]. Health insurance was based on the self-report of one or more months of health insurance over the past 12 months at Wave III. Additional details of the measures and responses are shown in Additional file 1: Appendix Table A.

### Statistical analysis

All analyses were performed using Stata version 15, and statistical significance thresholds were set at two-sided alpha = 0.05. We utilized pre-constructed sample weights provided by the National Longitudinal Study of Adolescent to Adult Health for all analyses to yield nationally representative estimates. Descriptive statistics were used for demographic and health care utilization characteristics, with chi-squared tests and t-tests to compare attributes of young adults with and without a physical disability. Univariate logistic regression was used to compare the number of hospital, ED, and mental health facility visits based on disability status. Multiple logistic regression was used to predict health care utilization controlling for a priori covariates, which included age, sex, race/ethnicity, insurance status [7], household income, highest parent education, and history of depression [31], asthma, diabetes, or seizure disorder.

## Results

Of the 13,436 participants that met inclusion criteria, 4.2% had a physical disability and 95.8% did not, using national weighting in our sample (Table 1). Women represented 50% of the sample and a majority of the sample was white. The average age at baseline was 15.9 years (standard error [SE] = 0.12), and at follow-up was 21.8 years (SE = 0.12). The median household income was similar between those with a disability ($41,000, inter-quartile range [IQR]: $26,000-$52,100) and those without ($42,070, IQR: $28,000-$53,900); mean and standard error values are reported in Table 1. Although we noted only few statistically significant demographic differences between those with a physical disability and those without, we found numerous differences in health characteristics. Those with a physical disability more frequently had depression (16.1% vs 11.6%, *p* = 0.02), asthma (21.2% vs 16.7%, *p* = 0.03), and diabetes (2.5% vs 0.9%, p = 0.003), though there was no difference in seizure disorder (1.5% vs 1.5%, *p* = 0.99). The two groups had a similar health insurance status at follow up (80.3% vs. 81.6%, *p* = 0.65).

**Table 1:** Demographic and health characteristics of US adolescents by disability status

	No Physical Disability^a^	Physical disability^a^	*p*
	*n* = 12,825	*n* = 611	
**Demographic characteristics**			
Age, mean (SE)^b^	21.8 (0.12)	22.0 (0.14)	0.12
Sex (%)			0.24
Female	49.6	53.0	
Male	50.4	47.0	
Race^c^ (%)			
White	77.3	82.4	0.04
Black/African American	16.9	13.8	0.13
Asian/Pacific Islander	4.8	3.5	0.30
Native American	4.8	6.1	0.26
Hispanic (%)	11.0	8.2	0.08
Household income, mean (SE)^b, d^	46.2 (1.4)	45.0 (2.4)	0.62
Parent highest education (%)			0.51
High school or less	33.8	35.8	
More than high school	66.2	64.2	
**Health characteristics**			
Depression diagnosis (%)	11.6	16.1	0.02
Asthma (%)	16.7	21.2	0.03
Diabetes (%)	0.9	2.5	0.003
Seizure disorder (%)	1.5	1.5	0.99
**Health care characteristics**			
Health insurance ≥ 1 month past year (%)	81.6	80.3	0.65
Health care utilization			
Physical past year (%)	34.8	39.5	0.14
Unmet health care needs past year (%)	22.1	29.8	0.002
Hospitalization past 5 years (%)	26.0	33.9	0.002
≥ 2 ED visits past 5 years (%)^e^	34.3	43.1	0.002
Mental health admission past 5 years (%)	1.9	1.7	0.69

All means and percentages are calculated with weighted data to reflect the representative proportion of the target US population

^a^Physical disability defined at baseline as: 1) difficulty using limbs; 2) using assistive devices such as a walker or wheelchair; or 3) using a brace or artificial limb

^b^SE = standard error

^c^Participants can select more than one race; percentages may not add to 100%

^d^Income measured in thousands of dollars

^e^ED = emergency department

A total of 65% of the sample had significant ED utilization, a hospital admission, a mental health facility admission, or any combination of these. Those with a physical disability (vs. without a physical disability) had a greater burden of unmet health care needs in the last year (29.8% vs. 22.1%, *p* = 0.002). Hospitalizations (33.9% vs. 26.0%, *p* = 0.002) and ED visits (43.1% vs. 34.3%, *p* = 0.002) in the past five years were also more common for those with a physical disability vs. without a physical disability. There were no statistically significant differences in routine physicals in the past year (39.5% vs. 34.8%, *p* = 0.14) or mental health admissions in the past five years (1.7% vs 1.9%, *p* = 0.69).

### Routine health care engagement

Our logistic regression models examining unmet health care needs and attending a routine physical in the past year are shown in Table 2, with adjustments for covariates. Those with a disability had higher odds of unmet needs than their peers without a physical disability (OR 1.41 [95% confidence interval {CI} 1.07 – 1.87]). By contrast, no statistically significant differences were observed between young adults with a physical disability and those without in the model predicting routine physical attendance (*p* = 0.18).

**Table 2:** Associations between adolescent physical disability status and young adult healthcare utilization ages 18–26 (*n* = 13,416)

	Physical disability (predictor)^a^	
	Adjusted OR (95% CI)^b^	*p*
Yearly physical	1.20 (0.92—1.57)	0.18
Unmet healthcare needs	1.41 (1.07—1.87)	0.02
ED visit ≥ 2^c^	1.34 (1.06—1.70)	0.02
Hospitalization	1.36 (1.07—1.72)	0.01
Mental Health Facility Admission	0.68 (0.31—1.51)	0.34

^a^Reference: no physical disability reported at baseline

^b^OR = odds ratio; CI = confidence interval; adjusted for age, sex, race/ethnicity, insurance, household income, parent highest education, depression, asthma, diabetes, seizure disorder

^c^ED = emergency department

### Emergency department visits and hospital admissions

Table 2 shows the results of our logistic regression models predicting various types of hospital visits according to physical disability status, with adjustments for covariates. Young adults with disabilities had higher odds of ≥ 2 ED visits (OR 1.34 [95% CI 1.06 – 1.70]) and hospitalization (OR 1.36 [95% CI 1.07 – 1.72]) compared to those without physical disabilities. However, there was no statistically significant difference in odds of mental health facility admission (*p* = 0.34).

## Discussion

This study is one of the first to examine long-term (over seven years) health care utilization among adolescents with physical disabilities transitioning to adult care. We found that, despite having similar exposure to routine physician appointments as those without physical disabilities, young adults with physical disabilities were more likely to present to the ED or be admitted to the hospital. We also found that this group had more unmet health care needs.

Despite similar engagement with primary care providers, young adults with physical disabilities more often used health care of last resort, such as ED or inpatient admission, when compared to counterparts without disabilities. This outcome aligns with prior studies, which found that adults with disabling conditions from childhood have nine times higher hospitalization rates compared to the general population [32]. These encounters likely represent a mix of deferred preventive care visits and emergent problems. Although the number of routine physician visits did not differ by disability status, frequency of these visitsmay not reflect adequacy of preventive care, as prior studies found reduced rates of screening among people with disabilities [33–35], sometimes well below those recommended by the US Preventive Services Task Force [10]. Lower preventive care among those with a physical disability may be due to personal factors (anxiety), social factors (poor communication with providers, feeling of inadequate support), provider factors (prioritizing acute problems over chronic comorbid conditions), or environmental factors (inaccessible exam tables and other physical access barriers) [36–38].

Young adults with physical disabilities were also more likely to have unmet health care needs; this was independent of insurance status, which is strongly associated with foregoing care [7, 39]. This confirms previously published findings [8, 12, 13, 39, 40] in a nationally representative, longitudinal sample, underscoring the importance of eliminating barriers in accessing care. While addressing provider communication and environmental factors, care teams should also consider those that lead to a feeling of helplessness. As suggested by a prospective study of adolescents with long-term illness, parent involvement, promotion of health self-efficacy, and early meeting with the adult team were associated with better mental wellbeing and satisfaction with adult care [41]. An emphasis on these social factors may empower patients to more readily seek medical advice.

Both unmet needs and utilization of last-resort care may be mitigated by development of a robust framework for the transition from pediatric to adult care, as previously shown in shorter-term studies [4]. This handoff challenge spans a diverse array of chronic childhood conditions, from sickle cell anemia to spina bifida [42, 43]. Young adults with disabilities are already at higher risk of unemployment, low socioeconomic status, and worse health outcomes compared to those without disabilities [10, 13, 44, 45]. Numerous interventions aim to smooth the transition, with varying degrees of success. One systematic review of transition interventions found that patient education and dedicated transition clinics with pediatric and adult providers were key ingredients to success [17]. Such jointly run clinics, sometimes referred to as “overlap” clinics, may reduce the effect of care transitions for adolescents with physical disabilities. Pediatricians are essential to a proper transition to adult care and should identify adolescents with physical disabilities who may benefit from additional support in the transition. Pediatricians may refer adolescents with disabilities to transition clinics often run by specialties, such as adolescent and young adult medicine or combined medicine-pediatrics providers, or follow published guidelines to support the transition of care [14, 46]. On the receiving end, adult primary care providers accepting new young adults should ensure a thorough handoff from the referring pediatric provider and offer transition clinic support to their patients as part of assuming their care. Well-executed transitions require complex coordination but show promise in improving adherence, increasing perceived health and self-care, decreasing hospitalization rates, and even potentially reducing health care costs [47, 48].

Findings from this study should be viewed in context of several limitations. First, “physical disability” was defined using clear questions, but data about underlying medical diagnoses was not available. It is possible that misclassification of physical disability status occurred; this would most likely be non-differential and biased toward the null hypothesis. Second, considering that the outcomes are self-reported, recall bias is also possible when measuring utilization of health care of last resort. It was not possible to verify the number of hospital admissions reported using the electronic medical record. Here again, non-differential misclassification of the outcome would likely bias toward the null hypothesis. Third, neither physical disability status nor the outcome variables of interest were recorded in Waves IV and V, limiting our ability to control for those variables or measure longer-term outcomes and transitions. Fourth, residual confounding is always a possibility in cohort studies, even though we controlled for important variables such as age, sex, race/ethnicity, insurance status, and major chronic conditions. Future research could investigate differences by type of insurance (e.g., public or private). Despite these limitations, key strengths of the study include using a prospective cohort design of a large, nationally representative sample of US young adults with a follow-up interval of seven years.

In conclusion, this large, nationally representative study of adolescents transitioning to young adulthood found that those with a childhood physical disability are more likely to have unmet health care needs and to use last resort health care, compared to their peers without disabilities. In the context of similar engagement with preventive care, this finding indicates a need for the improved quality, not quantity, of health care interactions with young adults with disabilities. Future interventions should continue to emphasize multidisciplinary transition clinics, social support, and caregiver engagement throughout this critical transition. New options to optimize health care utilization among young adults with disabilities include peer navigators [49] and youth stakeholder participation and shared decision making for youth-friendly health services [50].

## Supplementary Information

**Additional file 1: Appendix Table A.** Variable definitions in the National Longitudinal Study of Adolescent to Adult Health.

## Data Availability

This study analyses restricted-use data from Add Health. Persons interested in obtaining Data Files from Add Health should contact Add Health, The University of North Carolina at Chapel Hill, Carolina Population Center, 206 W. Franklin Street, Chapel Hill, NC 27,516–2524 (http://www.addhealth_contracts@unc.edu). Further information on how to obtain the Add Health data files is available on the Add Health website (http://www.cpc.unc.edu/addhealth). The authors did not receive special access privileges to the data that others would not have. This study and its procedures were approved by the University of North Carolina Institutional Review Board. Written informed consent was obtained from all study participants (if over age 18) and/or their parents (if under age 18). All methods were carried out in accordance with the Declaration of Helsinki.

## Abbreviations

CI: Confidence interval
ED: Emergency department
OR: Odds ratio
SE: Standard error
US: United States
