# Supplementary material for: Health care utilization in young adults with childhood physical disabilities: a nationally representative prospective cohort study
Source: BMC Pediatr. 2022 Aug 25;22:505. doi: 10.1186/s12887-022-03563-0 (PMC9413894; doi:10.1186/s12887-022-03563-0)
Supplement: Supplementary file 1 — Additional file 1: Appendix Table A. Variable definitions in the National Longitudinal Study of Adolescent to Adult Health. [file 12887_2022_3563_MOESM1_ESM.docx]

| Appendix Table A: Variable definitions in the National Longitudinal Study of Adolescent to Adult Health | | | |
| --- | --- | --- | --- |
| **Variable** | **Question(s) Used** | **Timing of Question** | **Interpretation of Response** |
| Physical disability | Do you have difficulty using your hands, arms, legs, or feet because of a physical condition? Do you use a brace, cane, crutches, walker, medically prescribed shoes, wheelchair, or scooter because of a physical condition? Do you use an artificial hand, arm, leg, or foot? | Baseline (Wave I) | Dichotomized: at least one affirmative = yes, no to all = no |
| Household income | About how much total income, before taxes did your family receive in 1994? Include your own income, the income of everyone else in your household, and income from welfare benefits, dividends, and all other sources. | Baseline (Wave I), parent survey | Numeric, thousands of US dollars |
| Parent highest education | How far did your current (spouse/partner) go in school? How far in school did your biological father go? | Baseline (Wave I), parent survey | Dichotomized: high school or less vs. more than high school |
| Depression | Have you ever been diagnosed with depression? | Follow-up (Wave III) | Yes/no |
| Asthma | Have you ever been diagnosed with asthma? | Follow-up (Wave III) | Yes/no |
| Diabetes | Have you ever been diagnosed with diabetes? | Follow-up (Wave III) | Yes/no |
| Seizure disorder | Have you ever been diagnosed with epilepsy or seizure disorder? | Follow-up (Wave III) | Yes/no |
| Health insurance | Over the past 12 months, how many months did you have health insurance? | Follow-up (Wave III) | Dichotomized: at least one = yes, zero = no |
| Attendance of annual physical | When did you last have a physical exam or routine check-up? | Follow-up (Wave III) | Dichotomized: within the last 12 months = yes, any other response = no |
| Unmet health care needs | Has there been any time in the past 12 months when you thought you should get medical care, but you did not? | Follow-up (Wave III) | Yes/no |
| Significant ED usage | In the past five years, how many times have you been seen in an emergency room or ER? | Follow-up (Wave III) | Dichotomized: at least twice = yes, one or none = no |
| Hospital admission | In the past five years, how many times have you been hospitalized--that is, admitted to the hospital for at least a one-night stay? | Follow-up (Wave III) | Dichotomized: at least once = yes, zero = no |
| Mental health facility admission | In the past five years, have you spent a day or more in a facility where you were treated for a mental illness? | Follow-up (Wave III) | Yes/no |
